# Analgesic effect of neuromodulation using the AT-04 portable magnetic field-generating device in a patient with neuropathic pain: a case report

**DOI:** 10.1186/s40981-024-00694-4

**Published:** 2024-02-10

**Authors:** Atsushi Sawada, Michiaki Yamakage

**Affiliations:** Department of Anesthesiology, Sapporo Medical University School of Medicine, South 1, West 16, Chuo-Ku, Sapporo, 060-8543 Japan

**Keywords:** Neuromodulation, Magnetic field exposure, Neuropathic pain

## Abstract

**Background:**

Neuromodulation by magnetic field through the AT-04 (ait® (AT-04); Peace of Mind Co., Ltd., Kumamoto, Japan) has improved allodynia in neuropathic pain model rats. This report focuses on neuromodulation through magnetic field exposure using the AT-04 that provided an analgesic effect in a patient with neuropathic pain.

**Case presentation:**

A 47-year-old man presented with flaccid paralysis and extensive neuropathic pain and scored 7 on the 11-point Numerical Rating Scale (NRS) for his left upper limb. The patient was treated with neuromodulation by magnetic field exposure using the AT-04. Baseline NRS scores were obtained three times daily during the baseline period (days 1–5). Magnetic field exposure was then performed for 30 min three times daily (morning, noon, and evening) at home for 36 days, which was termed the intervention period (days 6–41). During the baseline period, the median NRS score was 7 and the baseline NRS score for calculating the percentage of nonoverlap data (PND) was 6. During the intervention period, the median NRS score was 4 and the PND value of the NRS score was 77.8% (28/36). Neuromodulation by magnetic field exposure using the AT-04 effectively decreased the patient’s NRS score. The patient had no adverse effects during the intervention period.

**Conclusions:**

Neuromodulation by magnetic field exposure using the AT-04 was effective in decreasing the NRS score in a patient with neuropathic pain. The AT-04 portable magnetic field-generating device shows potential as a therapeutic option for refractory neuropathic pain.

## Background

The neuromodulation produced by electrical therapies such as transcutaneous electrical nerve stimulation (TENS) and spinal cord stimulation (SCS) can alleviate neuropathic pain [1, 2]. A previous clinical trial has reported that neuromodulation through magnetic field exposure provided analgesic effects in patients with fibromyalgia [3]. It was recently reported that a portable magnetic field-generating device (ait® (AT-04); Peace of Mind Co., Ltd., Kumamoto, Japan) improved allodynia in neuropathic pain model rats via the endogenous pain modulation systems, including both the descending pain modulatory system and the opioid analgesic system [4]. The present study reports the case of a patient with neuropathic pain in whom neuromodulation through magnetic field exposure through the AT-04 led to a reduction in pain.

## Case presentation

**Fig. 1:**
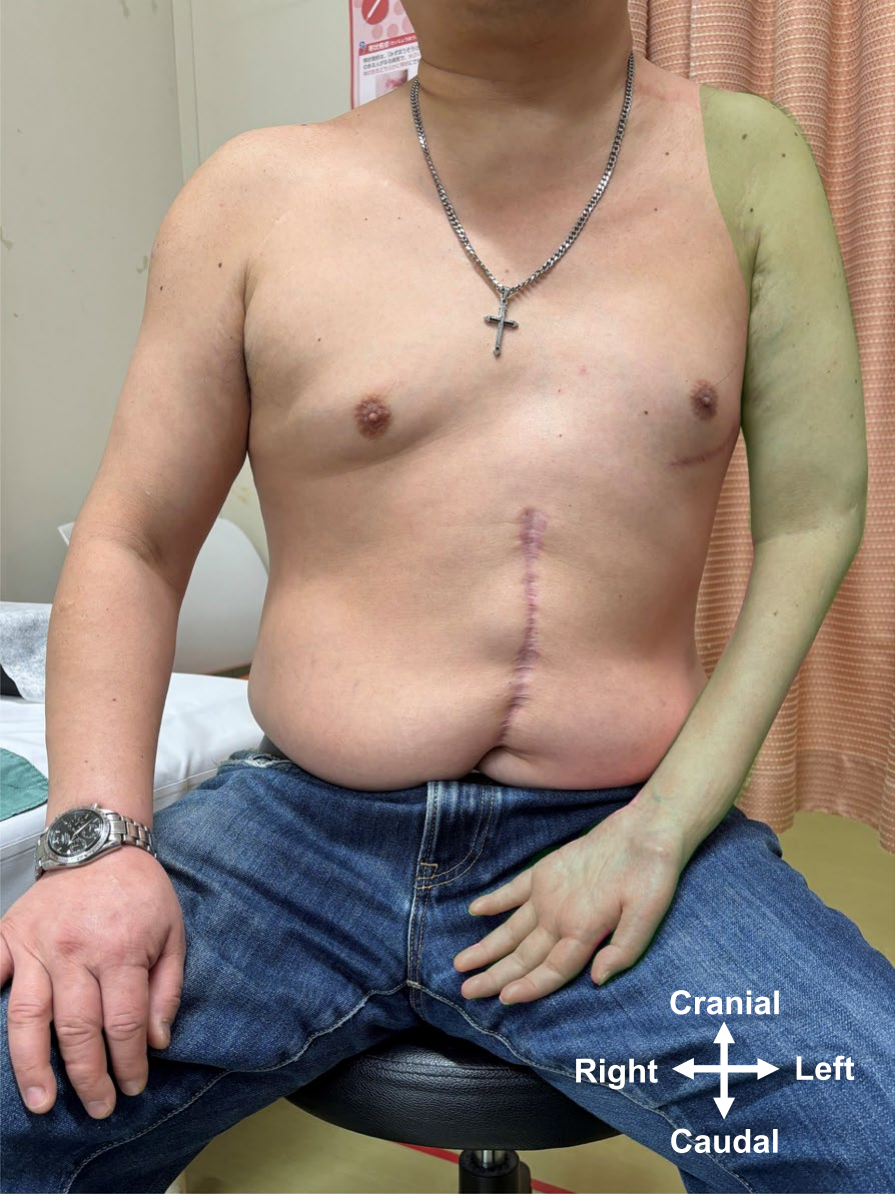
The patient presented with extensive neuropathic pain, flaccid paralysis, and complete sensory loss, but without allodynia, in the whole left upper limb from the shoulder to the hand (area colored green)

**Fig. 2:**
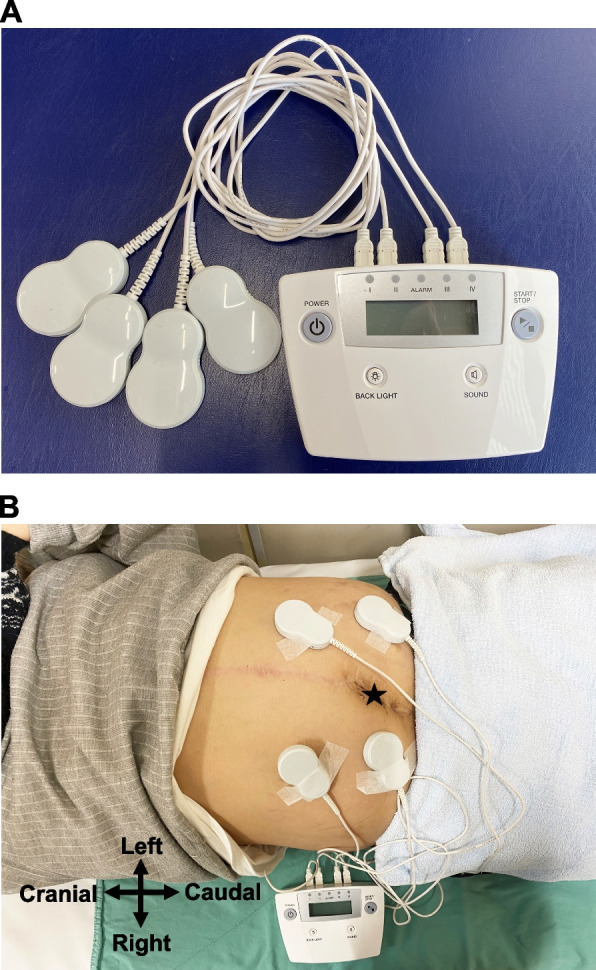
The AT-04 portable magnetic field-generating device. **A** Photograph of the device, which consists of a controller and four dual-coil emitters. **B** Four dual-coil emitters are applied to the abdomen around the patient’s umbilicus. The scar is from a previous abdominal surgery. The black star indicates the umbilicus

**Fig. 3:**
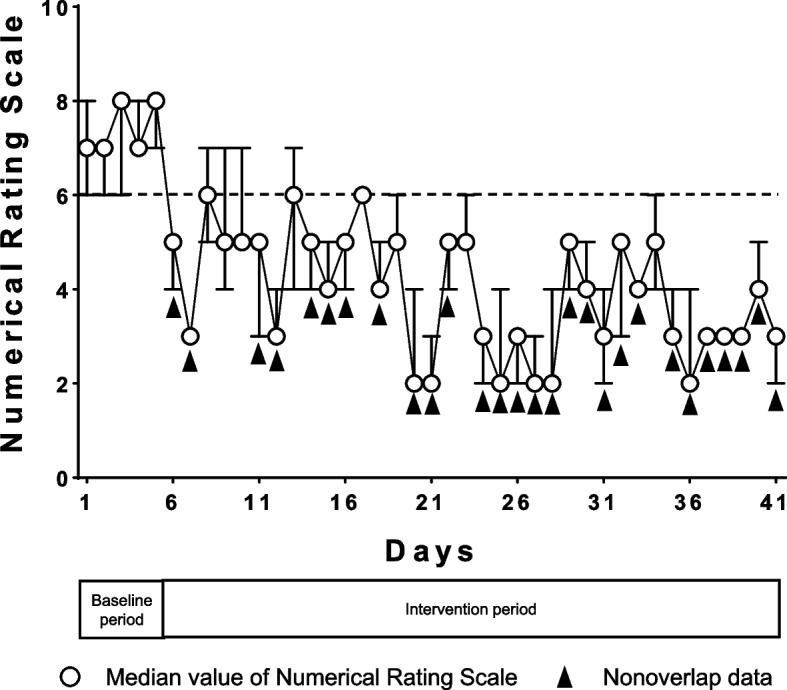
Numerical Rating Scale (NRS) scores during the baseline and intervention periods. The white circles indicate the median values, and the upper and lower bars indicate the minimum and maximum values, respectively. Black dotted line: reference data of the baseline NRS score used for calculating the percentage of nonoverlap data. Black arrowheads indicate nonoverlap data during the intervention period

A 47-year-old man (weight, 78 kg; height, 168 cm; body mass index, 27.6 kg/m^2^) was referred to our hospital’s pain clinic with a 9-year history of left brachial plexus avulsion injury that was sustained in a motorbike accident. He had previously undergone abdominal and left upper limb surgeries. He presented with extensive neuropathic pain, flaccid paralysis, and complete sensory loss, but without allodynia, in the whole left upper limb from the shoulder to the hand (Fig. 1). The Numerical Rating Scale (NRS) score for his left upper limb was 7 on the 11-point scale. Regional anesthesia, including cervical epidural anesthesia and brachial plexus block, had no analgesic effect on his neuropathic pain. Treatment with oral mirogabalin (30 mg/day), duloxetine (40 mg/day), and a fentanyl patch (6 mg/day) did not adequately alleviate the pain. We treated the patient with neuromodulation by magnetic field exposure using an AT-04 device, in addition to these pharmacological treatments. The AT-04 is a portable magnetic field-generating device comprising a controller and four dual-coil emitters (Fig. 2A). The dual-coil emitters simultaneously generate alternating magnetic fields at 2 kHz and 83.3 MHz, with field strengths of 20–30 μT and 400–700 nT, respectively. The overall energy produced by the magnetic fields generated by the AT-04 is approximately one-third of terrestrial magnetism [3, 4]. We applied four dual-coil emitters around the patient’s umbilicus (Fig. 2B). Because he lived alone, he could not place the dual-coil emitters on his left upper limb by himself. Baseline NRS scores were obtained three times daily during the baseline period (days 1–5). Magnetic field exposure was then performed at home for 30 min three times daily (morning, noon, and evening) for 36 days, which was termed the intervention period (days 6–41). The NRS score was obtained after each exposure session during the intervention period. Adverse effects were self-recorded by the patient during the intervention period. NRS scores are presented as the median value with the minimum and maximum values (Fig. 3) and were analyzed using the percentage of nonoverlap data (PND) in single-subject research (Table 1) [5]. During the baseline period, the median NRS score was 7 and the baseline NRS score for calculating the PND was 6. During the intervention period, the median NRS score was 4 and the PND value of the NRS score was 77.8% (28/36) (Fig. 2). Neuromodulation by magnetic field exposure through the AT-04 effectively decreased the patient’s NRS score. The patient had no adverse effects during the intervention period. Treatment by magnetic field exposure using the AT-04 was discontinued after the intervention period at the request of the patient due to the economic burden of the rental fee for the AT-04 device (approximately 20,000 Japanese yen per month). The NRS score of his neuropathic pain was 7 at day 28 after stopping treatment by magnetic field exposure using the AT-04.

**Table 1:** Criteria for the percentage of nonoverlap data (PND) for interventional effectiveness [5]

PND value	Interventional effectiveness
PND ≥ 90%	Very effective treatment
70% ≤ PND < 90%	Effective treatment
50% ≤ PND < 70%	Questionable treatment
PND < 50%	Ineffective treatment

## Discussion

We present the case of a patient with neuropathic pain in whom neuromodulation via magnetic field exposure using the AT-04 achieved a decrease in his NRS score. This is the first clinical report of this effect in a patient with neuropathic pain. Recommended pharmacotherapies for neuropathic pain include gabapentinoids, serotonin noradrenaline reuptake inhibitors (SNRIs), and opioids, which can have the side effects of sedation, dizziness, nausea, vomiting, and constipation [6, 7]. Neuromodulation by electrical therapies such as TENS and SCS can alleviate neuropathic pain [1, 2] but is accompanied by uncomfortable pulsations and painful sensations. In addition to its analgesic effect, the AT-04 device has the advantage of producing no meaningful sensory discomfort in patients, which suggests its potential as a treatment option for patients with refractory neuropathic pain.

The detailed mechanisms underlying the analgesic effect of neuromodulation via magnetic field exposure using the AT-04 remain unclear. Kohno et al. reported an analgesic effect of the AT-04 by magnetic field exposure when it was applied in the vicinity of the spinal cord and abdomen as well as to the affected part of the body [4]. In the present case, magnetic fields generated by emitters placed around the patient’s umbilicus effectively decreased the NRS score of neuropathic pain in the patient’s left upper limb. A previous study has suggested that magnetic fields have the effect of activating cellular functions that promote regeneration and repair of peripheral sensory nerves [8] by activation of both the descending pain modulation system and the endogenous opioid analgesic system [4]. The range of the magnetic fields generated by the emitters placed around the patient’s umbilicus reached the whole body including the spinal cord and the left upper limb. Accordingly, we consider that neuromodulation through magnetic field exposure using the AT-04 might have relieved neuropathic pain in the present patient via the activation of both the descending pain modulation system and the endogenous opioid analgesic system.

There are some limitations in the present case report. First, we did not investigate whether the analgesic effect of neuromodulation through magnetic field exposure using the AT-04 would provide an additive effect on background pharmacotherapy in patients taking SNRIs and opioids. Second, we did not clarify the optimal time per session or frequency per day of magnetic field exposure using the AT-04. Third, in the present case, neuromodulation through magnetic field exposure using the AT-04 decreased the NRS score of neuropathic pain but did not lead to a reduction in dose of the pharmacological treatments.

We presented a case of neuropathic pain in which neuromodulation through magnetic field exposure using the AT-04 successfully decreased the patient’s NRS score. The AT-04 portable magnetic field-generating device has potential as a therapeutic option for refractory neuropathic pain.

## Data Availability

The dataset supporting the conclusions of this article is included within the article.

## Abbreviations

TENS: Transcutaneous electrical nerve stimulation
SCS: Spinal cord stimulation
NRS: Numerical Rating Scale
PND: Percentage of nonoverlap data
SNRI: Serotonin noradrenaline reuptake inhibitors
